# Novel Analysis Methodology of Cavity Pressure Profiles in Injection-Molding Processes Using Interpretation of Machine Learning Model

**DOI:** 10.3390/polym13193297

**Published:** 2021-09-27

**Authors:** Jinsu Gim, Byungohk Rhee

**Affiliations:** 1 Department of Chemical Engineering, Hanyang University, 55 Hanyangdeahak-ro, Ansan 15588, Korea; interactionjs@gmail.com; 2 Department of Mechanical Engineering, Ajou University, 206, Worldcup-ro, Suwon 16499, Korea

**Keywords:** injection molding, cavity pressure, interpretable machine learning

## Abstract

The cavity pressure profile representing the effective molding condition in a cavity is closely related to part quality. Analysis of the effect of the cavity pressure profile on quality requires prior knowledge and understanding of the injection-molding process and polymer materials. In this work, an analysis methodology to examine the effect of the cavity pressure profile on part quality is proposed. The methodology uses the interpretation of a neural network as a metamodel representing the relationship between the cavity pressure profile and the part weight as a quality index. The process state points (PSPs) extracted from the cavity pressure profile were used as the input features of the model. The overall impact of the features on the part weight and the contribution of them on a specific sample clarify the influence of the cavity pressure profile on the part weight. The effect of the process parameters on the part weight and the PSPs supported the validity of the methodology. The influential features and impacts analyzed using this methodology can be employed to set the target points and bounds of the monitoring window, and the contribution of each feature can be used to optimize the injection-molding process.

## 1. Introduction

Molding conditions in a cavity are the most important factors relating to product quality. However, it is difficult to observe the molding conditions such as cavity pressure and cavity surface temperature that are not directly measurable through the outer mold wall. Signals from an injection-molding machine (IMM) do not directly represent the molding conditions in the cavity because of the long process distance from IMM to the cavity and the high damping nature of the polymer melt [1]. A long melt delivery system extends the processing distance. The viscous and compressible characteristics of the polymer melt in the delivery system dampen the response of the molding conditions in the cavity to the operation of IMM.

To analyze the molding conditions in the cavity, in-mold sensors have been used as essential parts of process monitoring systems. Ageyeva et al. emphasized the importance of the process data measured by in-mold sensors for Industry 4.0 [2]. Zhao et al. regarded the sensing of the physical state of the process as one of the phases of intelligent injection-molding process [3]. Molding conditions can be measured using in-mold sensors installed on the cavity surface. For example, Gordon et al. employed cavity pressure sensors to measure the cavity pressure variation in the filling stage due to changes in barrel temperature or material characteristics [4]. Gim et al. applied temperature sensors to measure gradual increases in the cavity surface temperature due to the accumulation of heat within an injection mold [5]. By using multiple in-mold sensors, complex molding phenomena can be analyzed. Gao et al. measured melt velocity by using multivariate sensors [6]. Han et al. analyzed melt viscosity by using thermocouples and pressure sensors [7]. Friesenbichler et al. measured the pressure dependency of the shear viscosity using infrared temperature sensors and pressure sensors [8].

The use of in-mold sensors is important for optimizing injection-molding processes. Menges et al. indicated that the non-uniform performance of IMMs, variation of material characteristics, and the ambient conditions make the relationships between process parameters and quality unreliable [9]. Consequently, it has been suggested that evaluating molding conditions using in-mold sensors has been regarded as the most dependable method for the systematic optimization of injection-molding processes [10]. Therefore, it was recommended that the adjustment of process parameters to optimize the pressure or temperature profile of in-mold sensors [11]. To effectively optimize the injection-molding process using the in-mold sensor profile, the impact of each feature in the profile on the quality needs to be analyzed. However, this requires prior knowledge and understanding on the injection-molding process and polymer materials.

Special-purpose in-mold sensors have been developed. For example, Kim et al. developed venting sensors measuring the temperature and pressure elevation due to gas compression in the cavity have been proposed [12]. Gim et al. proposed an indirect pressure sensor installed below the lens core pin to measure the cavity pressure without any sensor marks on the optical surface [13]. Chen et al. devised a linear displacement transducer to measure the mold separation [14]. Debey et al. proposed the thermocouple array sensor to measure the temperature distribution in the thickness direction [15]. Capacitance sensors were used to measure the shear stress, and the flow length increase [16,17]. Because the special-purpose in-mold sensors are supposed to be used only for specific purposes, general-purpose cavity pressure sensors have been widely used instead.

**Figure 1:**
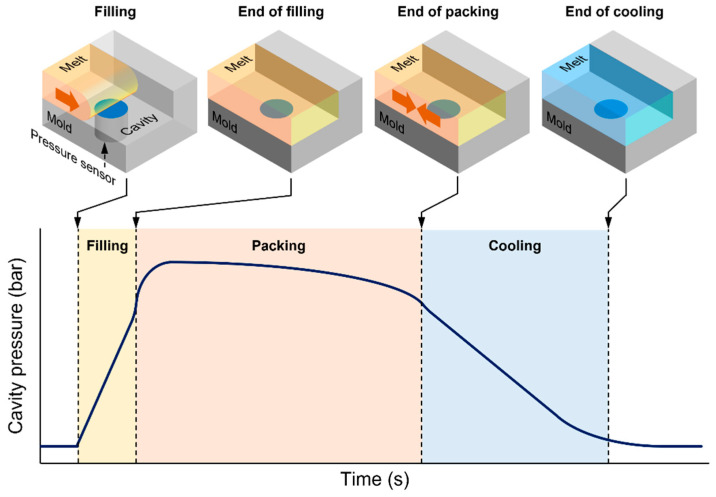
Typical injection-molding cavity pressure profile and process stages.

A typical cavity pressure profile of a cold runner mold represents three process stages of filling, packing, and cooling as shown in Figure 1. In each process stage, features of the cavity pressure profile can be related to the process conditions and part quality. For example, the melt viscosity and flow front speed increase the slope of the pressure profile in the filling stage. The packing pressure as a process parameter of IMM and the cooling conditions of the mold affect the plateau of the pressure profile during the packing stage. Over- or under-packing pressure of the packing stage influences the decay of the pressure profile in the cooling stage. The complicated interactions between the process parameters and the process stages make the interpretation of a cavity pressure profile highly dependent on personal experience and knowledge.

Analysis of variance (ANOVA) is widely used to check the effect of process parameters on part quality and to optimize the molding process [18,19]. Mehat and Kamaruddin analyzed the effect of process parameters on the flexural modulus of mold parts [20]. Altan applied Taguchi method and ANOVA to determine optimal process setting [21]. To apply ANOVA to investigate the effect of a cavity pressure profile, each feature in the profile should be controlled independently based on the design of experiment (DOE). Controlling each feature of the cavity pressure profile by adjusting the process parameters is difficult because the responses of the features are coupled to each other.

Machine learning has been used for quality prediction and optimization of injection-molding processes [19]. It requires less prior experience and knowledge to model relationships in the injection-molding process. Building a training dataset of the injection molding process is expensive and time-consuming. Ozcelik and Erzurumlu pointed out that the orthogonal array used for Taguchi method requires the minimum time and resources to get the information about the design parameters [22]. Oliaei et al. also indicated that orthogonal array is an efficient method for DOE [23]. Therefore, many researchers used an orthogonal array to build a training dataset [24]. Computer simulations have been proposed as a substitute for physical experiments or to build pre-trained models for transfer learning. Li et al. and Guo et al. applied genetic algorithm for neural network trained by moldflow simulation result to reduce warpage [25,26]. Shi et al. applied parametric evaluation strategy on artificial neural network trained by simulation result to optimize molding process [27]. Tercan et al. built a pre-trained model by the simulation result and applied transfer learning to the pre-trained model [28]. Lee et al. developed the process condition recommendation system using a pre-trained neural network model [29]. Process parameters have been used for optimization using the machine learning. Tsai and Luo selected mold temperature, cooling time, and packing time to be optimized [30]. Changyu et al. applied genetic algorithms on mold and melt temperature, injection and placing time, and holding pressure to optimization [31]. However, the process parameters on the machine side are not sufficient for optimization [9]. Therefore, it is desirable to make use of in-mold sensor signals with machine learning technology for optimizing injection-molding processes.

In this study, a novel analysis methodology for cavity pressure profiles is proposed. Process state points (PSPs) are extracted to represent the features of the molding conditions in the cavity. A simple neural network is trained to correlate the PSPs and part weight as a quality index. The trained neural network is interpreted to analyze the impact of each PSP on the part weight. The most influential PSPs are selected, and their impact are compared with the ANOVA results for process parameters to check the validity of the proposed methodology. The proposed methodology contributes to the systematic monitoring and optimization of injection-molding processes.

## 2. Methodology

Molding conditions in the cavity have a significant impact on part quality. In particular, the part weight and dimensions are directly affected by the pressure and temperature in the cavity due to the pressure–volume–temperature (PvT) characteristics of polymer materials. The pressure condition in the cavity can be represented by the cavity pressure profile of in-mold pressure sensor. The features in the cavity pressure profile have different effects on part quality. For example, the early part of the cavity pressure profile is important to the surface quality, which is primarily influenced by the filling condition [32]. Accordingly, the impact of the features on quality should be quantified to analyze the cavity pressure profile. These can be analyzed using the interpretation of a machine learning model as a metamodel representing the relationship between the cavity pressure profile and part quality.

**Figure 2:**
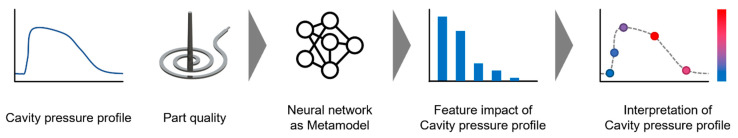
Schematic diagram of the proposed methodology.

Figure 2 shows a diagram of the methodology, which uses a conventional neural network model to build a metamodel. Feature points are extracted from the cavity pressure profile and used as input features for the neural network. The trained neural network is analyzed using an interpretable machine learning method. Consequently, the cavity pressure profile is interpreted based on the overall impact and contribution of each feature.

The methodology proposed in this study was verified using a typical cavity pressure profile and the well-known responses of part weight to the process parameters. The process parameters in the packing stage predominantly influence the part. Consequently, a comparison of the influence of process parameters with the results of the methodology was used to verify the proposed methodology. Chen and Turng suggested the part weight as a measure of the quality [33]. Therefore, the part weight was measured as the quality index because the part weight is easy to measure without complicated measurements and appropriate for lab-scale experiments.

## 3. Experiment

### 3.1. Polymer Material

High-impact polystyrene (HIPS) 60HR manufactured by LG Chem Ltd. (Seoul, Korea) was used for the molding trials. The HIPS was dried at 80 °C for 4 h using a vacuum dryer to prevent bubble formation and better metering during plasticization through the evaporation of moisture content.

### 3.2. Injection Mold and Molding Machine

**Figure 3:**
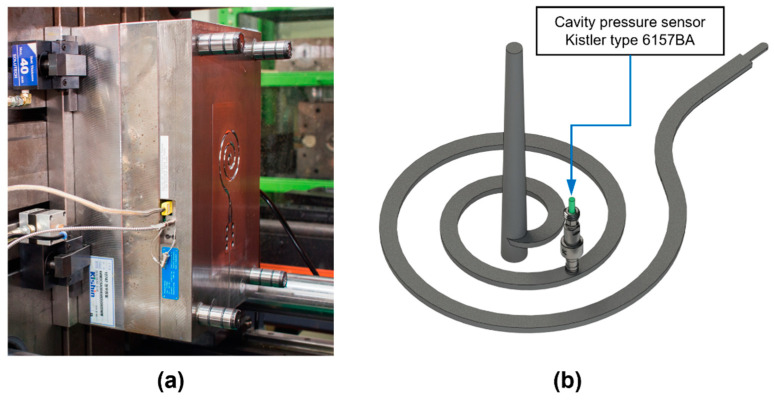
Injection mold setup (**a**) moving plate of spiral mold, and (**b**) cavity geometry and pressure sensor position.

A mold with a spiral cavity was used for the molding trials to measure a typical cavity pressure profile, as shown in Figure 3a. A spiral cavity of dimensions 5 × 3 × 485 mm (width × height × flow length) shown in Figure 3b was designed to be filled easily using an injection pressure of approximately 1500 bar. During the molding trial, the spiral cavity geometry was fully filled under 2000 bar of the maximum injection pressure. The long flow length resulted in a long time of the filling stage, and each molding stage is clearly shown in the cavity pressure profile. It made the mold appropriate for verification of the proposed methodology. The response of the cavity pressure profile to the process parameters can be easily explained owing to the simple cavity geometry and flow pattern.

The coolant used for the mold was maintained at 40 °C. A high-speed electric IMM (LGE150IIIDHS, LS Mtron Ltd., Anyang, Korea) with a clamping force of 150 tons was used. The screw diameter was 25 mm, and the maximum injection pressure was 3500 bar. The maximum injection speed was 1000 mm/s.

**Figure 4:**
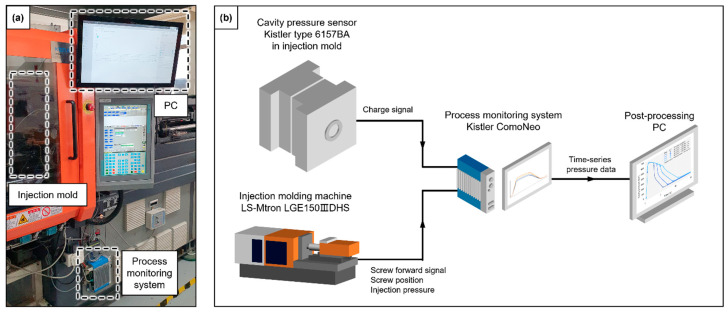
Process monitoring system setup (**a**) overall setup, and (**b**) signal connections.

A typical piezo-type cavity pressure sensor Type 6157BA (Kistler AG, Winterthur, Switzerland) was placed in the middle of the flow length to measure the cavity pressure, as shown in Figure 3b. The charge signal from the sensor was amplified using a Type 5887A ComoNeo process monitoring system (Kistler AG, Winterthur, Switzerland), as shown in Figure 4a. The start of the pressure measurement was synchronized to the screw forward signal from the IMM, as shown in Figure 4b. The screw position and injection pressure were measured to set the initial process parameters.

### 3.3. Experimental Conditions

The process parameters were selected based on their expected influence on the part weight. The packing pressure and time of the packing stage were expected to be the most influential factors on the part weight because the packing stage compensates for shrinkage of the polymer material. However, the injection speed was expected to be less influential than the packing pressure and time because the filling stage predominantly affects the surface quality, not shrinkage in the packing and cooling stages [34]. These are common process parameters that are tuned to optimize the injection-molding process. Each process parameter value was set to maintain a typical cavity pressure profile shown in Figure 1. For each process condition, optimum filling to packing (velocity to pressure, VP) switchover position was searched to eliminate a sharp peak of late VP switchover position and a sudden drop of early VP switchover position [35]. The selected process parameters and levels are listed in Table 1. The DOE was a full factorial design. To consider the process fluctuation, three specimens for each process condition were molded. The part weight including the runner was measured at a resolution of 5 mg. The data analysis software Minitab 16.2 (Minitab LLC, State College, PA, USA) was used to analyze the effect of the process parameters on the part weight and feature points of the cavity pressure profile. The effects of the process parameters were compared to verify the validity of the proposed methodology.

## 4. Modeling and Analysis

### 4.1. Process State Points (PSPs)

The cavity pressure profile was time-series data that was a set of time and pressure values. The quality of the time-series data depended on the performance of the data acquisition system. The number of data points varies based on the sampling rate, measuring time, and the variable sampling rate resulted in an irregular time interval between each data point. Maintaining a constant and high sampling rate required a high data acquisition performance. Using all the values of the time-series data for a neural network as input features was inefficient and required significant computing power.

**Figure 5:**
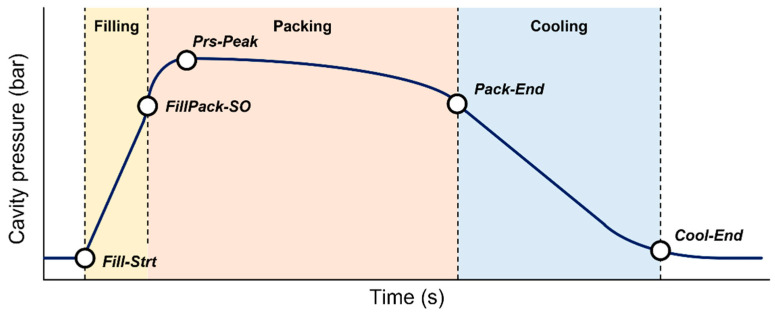
Process state points (PSPs) of typical cavity pressure profile and effective process stages in the cavity.

In a typical cavity pressure profile, the five feature points representing the characteristics of the molding condition could be extracted as PSPs. Each PSP divides the process stages. The start and end points of the filling, packing, and cooling stages were the four PSPs. The maximum cavity pressure representing the overall pressure level was the other PSP. The names and meanings of the PSPs are listed in Table 2 and shown in Figure 5. By extracting the PSPs from the cavity pressure profile, the problems of an irregular sampling rate and the number of data points can be resolved. Zhao and Gao proposed a similar approach to represent the melt temperature profile from the nozzle of IMM [36].

Referring to machine signals such as the injection pressure and screw position would not be appropriate for extracting the PSPs. The time of the start and end of the process stages is usually defined by machine signals. The process parameters and machine operation cannot represent the molding conditions in the cavity [1]. The cavity pressure would not respond immediately to the machine operation due to the long melt delivery system and damping effect of the viscosity. Consequently, it would be more appropriate to extract the PSPs from the in-mold sensor signals without the machine signals, except at the start and end of the process.

**Figure 6:**
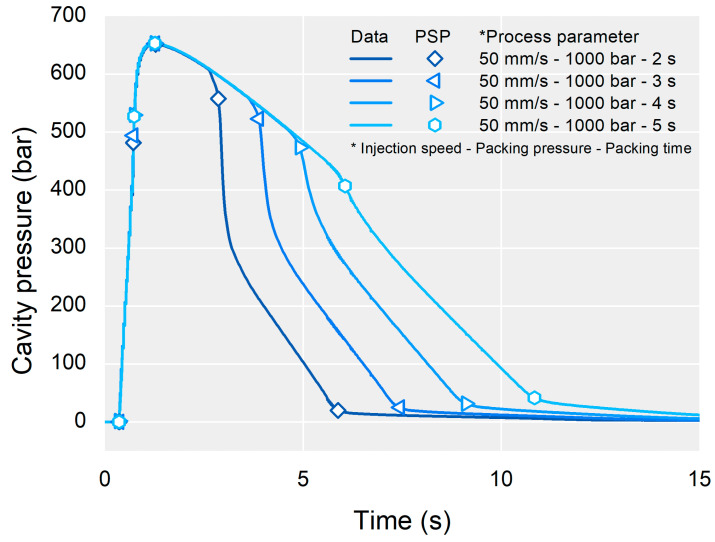
Example of the extraction of the process state points (PSP).

The segmentation algorithm of time-series data using perceptually important points developed by Chung et al. [37] was used to extract the PSPs. The algorithm determines the feature points of the cavity pressure profile shown in Figure 5. In the cavity pressure profile, two PSPs were extracted between the initial and peak pressures, and another two PSPs were extracted between the peak and end pressures. Five PSPs including the peak pressure were extracted as the features of the cavity pressure profile, as shown in Figure 6.

### 4.2. Neural Network Model

The relationship between the cavity pressure profile and part quality could be modeled using a neural network as a metamodel. The PSPs as features of the molding conditions in the cavity were closely related to part quality. Consequently, it was expected that a simple neural network could sufficiently represent the relationship. The machine signals and the pressure and temperature profile of the nozzle were expected to require a more complicated model because these signals were far from the actual molding conditions in the cavity.

**Figure 7:**
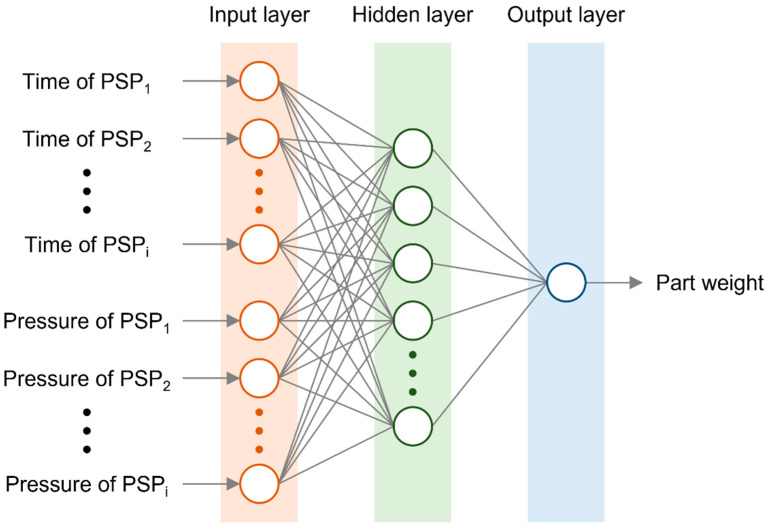
Structure of the multi-layer perceptron with fully connected neurons.

To build a neural network model, a multi-layer perceptron (MLP) with a single hidden layer was selected. Lockner and Hopmann suggested that one hidden layer having a single digit number of neurons would be suitable for high model quality for injection molding [38]. The time and pressure values of the PSPs were inputs to the input layer of the MLP, and the part quality was predicted from the output layer, as shown in Figure 7. The architecture of the MLP is shown in Table 3. The time and pressure values were normalized using the cycle time and maximum injection pressure of IMM to prevent unstable training because of the differences in the order of values. The part weights were not normalized because the actual distribution range could not be specified. The data were randomly split into two sets. The data for training and validation was 80%, and the data for test was 20% of the dataset.

The neural network was implemented using Python version 3.7, TensorFlow version 2.2.0 and Keras version 2.3.1. The training was carried out on a laptop environment of Intel i7-9750H (CPU), 32 GB RAM, and Nvidia GTX1650 (GPU) running CUDA version 11.0.

### 4.3. Analysis of Neural Network Model

A neural network is usually called a “black box” because the meaning of a lot of weight and bias values composing the neural network cannot be directly interpreted. Understanding the model’s behavior is important for further application such as feature engineering and parameter tuning [42]. Kashyap and Datta pointed out that interpretation of neural network for injection molding process was not possible [43]. To understand the neural network structure of the injection molding process, Zhou et al. tried to extract influential features from the injection pressure of IMM using the sparse autoencoder method [44]. However, quantitative interpretation of the neural network was still difficult. Several methods have been suggested for interpreting neural networks recently. The Shapley Additive Explanations (SHAP) method can explain the reasons for prediction results using Shapley values of game theory [45,46]. The contribution to the prediction results of each input feature is quantified by the SHAP value in the same unit of the predicted value. Consequently, the cavity pressure profile can be interpreted by analyzing the neural network using the SHAP method, allowing influential features in the cavity pressure profile to be determined. In this study, SHAP version 0.38.1 was used.

## 5. Results and Discussion

### 5.1. Model Quality

**Figure 8:**
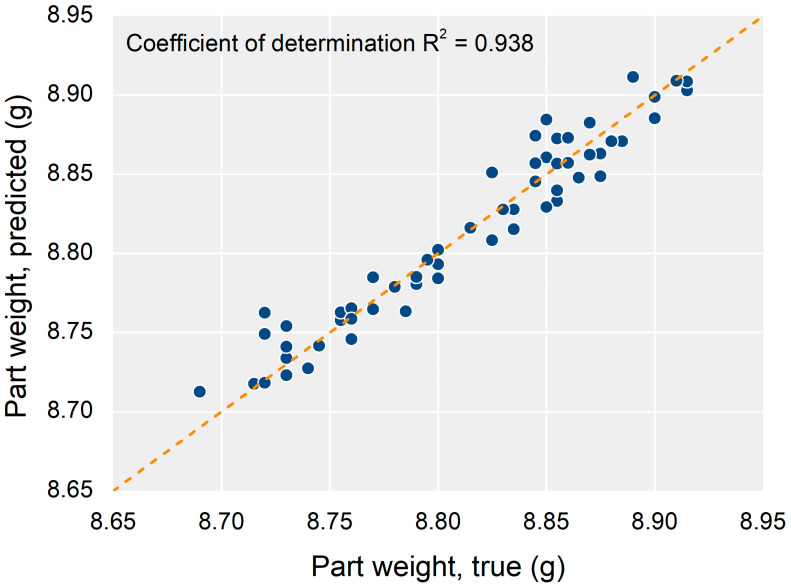
Part weight prediction result by the neural network.

The trained neural network exhibited good model quality representing the relationship between the PSPs and the part weight. The total training process required only 5 min the laptop environment. During the training, overfitting did not occur, and regularization was not required. The decrease in loss slowed down at approximately 8000 epochs, and therefore 10,000 epochs of the maximum number of training iteration were sufficient. A comparison between the true and predicted values is shown in Figure 8.

The coefficient of determination, *R*^2^, evaluates the quality of the model [47]. *R*^2^ is independent of the data scale, contrary to other performance indicators such as root-mean-square error (RMSE) or mean square error (MSE) [28]. *R*^2^ can be defined in Equation (1) as follows:

(1) $$R^{2}=1-\frac{\sum {\left(y_{i}-{\hat{y}}_{i}\right)}^{2}}{\sum {\left(y_{i}-{\bar{y}}_{i}\right)}^{2}}=\frac{SS_{ex}}{SS_{ov}},$$

where $SS_{ex}$ is the explained sum of squares, $SS_{ov}$ is the overall sum of squares, $y_{i}$ is the true value of the quality criterion, ${\hat{y}}_{i}$ is the prediction, and ${\bar{y}}_{i}$ is the average of the true values. The *R*^2^ of the model was approx. 0.94 for the test data, as shown in Figure 8. The neural network was trained stably, having a standard deviation of 0.01 for the *R*^2^ for 10 independently trained models from random data splitting.

Even though the neural network had only a single hidden layer, the part quality was predicted well by the neural network model because the cavity pressure profile was closely related to the part quality. This implies that the analysis of the trained neural network model could be regarded as an analysis of the actual relationship between the cavity pressure profile and part quality.

### 5.2. Interpretation of Cavity Pressure Profile

**Figure 9:**
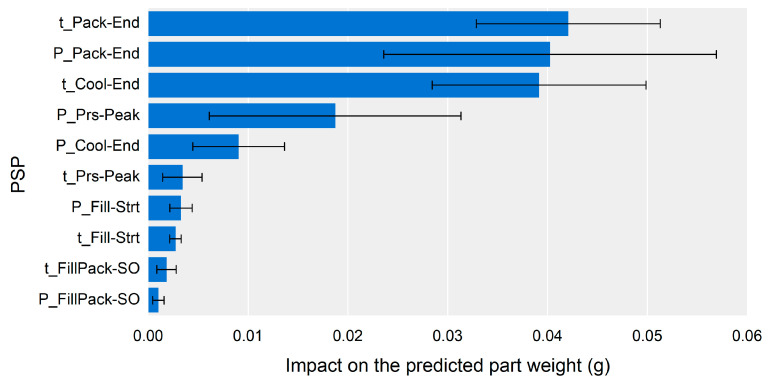
Impact of the PSPs to the part weight.

Figure 9 shows the SHAP analysis results for the trained neural network and the overall impact of each PSP on the part weight. The SHAP value indicates the contribution of each input feature to the prediction result. The impact was calculated by averaging the magnitudes of the SHAP values for all predictions.

The most influential feature was the time of Pack-End (t_Pack-End), followed by the pressure of the same PSP (P_Pack-End) and the time of the Cool-End (t_Cool-End). There were no significant differences between the top three influential features, as demonstrated by the overlapping error bars and mean points. This indicates that the time and pressure at the end of packing and the cooling end time were the most significant features to the part weight. The pressure at the peak point (P_Prs-Peak) was the next most influential PSP, and the other PSPs had less than half the impact of the top four PSPs.

**Figure 10:**
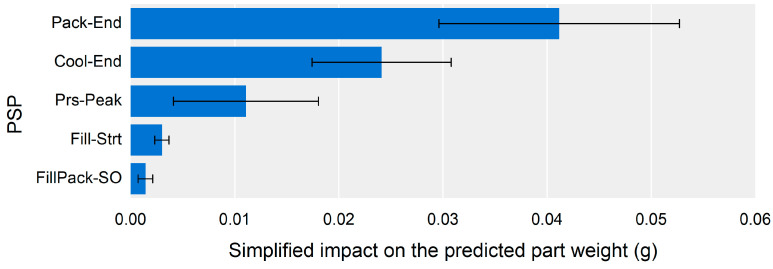
Simplified impact of the PSPs on the part weight.

The impact of a PSP was divided by the impact of its time and pressure values. As it is necessary to summarize the impact of time and pressure in a single value, a simplified PSP impact value could be defined by averaging the time and pressure impact. Figure 10 shows the simplified impact of the PSPs on the predicted part weight. The results made it easier to understand the impact order of the PSPs.

**Figure 11:**
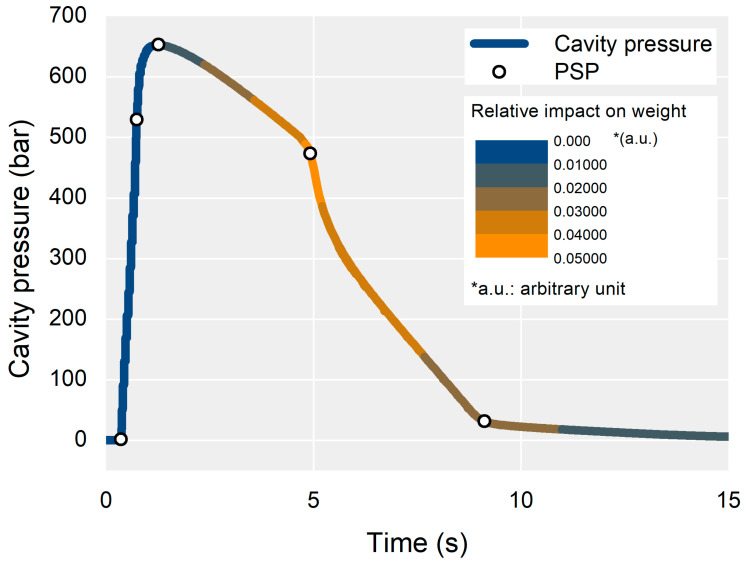
Impact mapping on the cavity pressure profile.

The most influential PSP on the part weight was Pack-End, followed by Cool-End and Prs-Peak. The impact of the PSPs could be represented by color mapping on the cavity pressure profile, as shown in Figure 11. The color of the cavity pressure profile was determined by interpolating the simplified impact of the PSPs. As shown in Figure 11, the most influential section of the cavity pressure profile on the part weight was near the Pack-End segment.

The impact of the PSPs on the part weight could be used to set the monitoring window for stable part quality. Figure 11 shows that the monitoring window should be set to the section from the pressure peak point to the endpoint of cooling. Figure 10 also shows that the target point for the monitoring window should be the PSPs of the Pack-End and Cool-End. The monitoring bounds for the PSPs can be set according to Figure 9. Controlling the most influential features was reasonable and practical in order to stabilize part quality. The time bounds of the PSP at the Pack-End and Cool-End, and the pressure bound of the PSP at the Pack-End to monitoring should be kept in a narrower range than the other time and pressure values of the PSP.

The SHAP analysis could explain the contribution of each input feature to the prediction results in an additive manner. The sum of the contribution represented in the SHAP value is the difference between the specific prediction result and the mean of all predicted values, as shown in Equation (2) below:

(2) $$P^{*}=\bar{P}+\sum C,$$

where C is the contribution of each time and pressure value of the PSP, $P^{*}$ is the specific prediction result, and $\bar{P}$ is the base value of the mean of all predicted values. If the neural network represented the actual relationship well, $P^{*}$ and $\bar{P}$ could be regarded as the actual values. Therefore, the SHAP analysis result for the specific sample shows the contribution of each PSP to the actual part weight.

**Figure 12:**
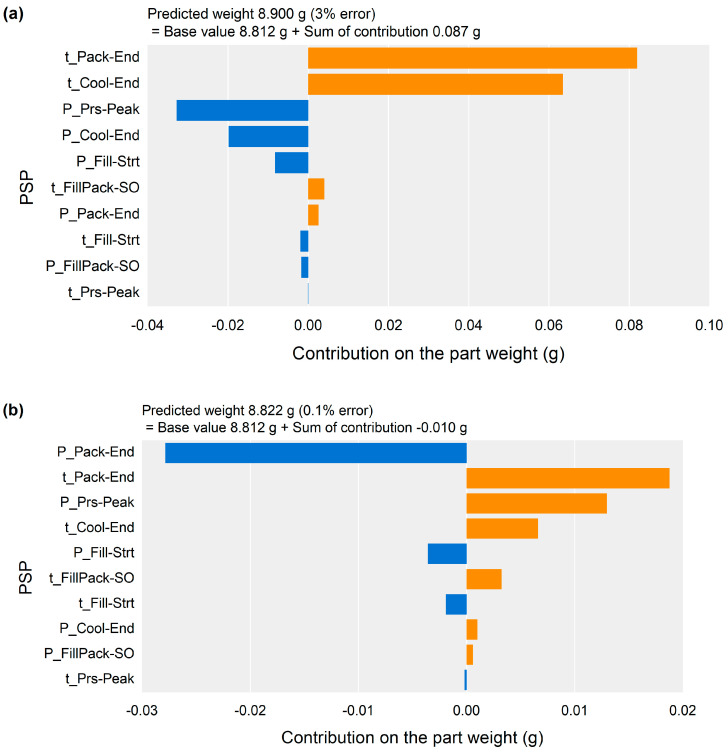

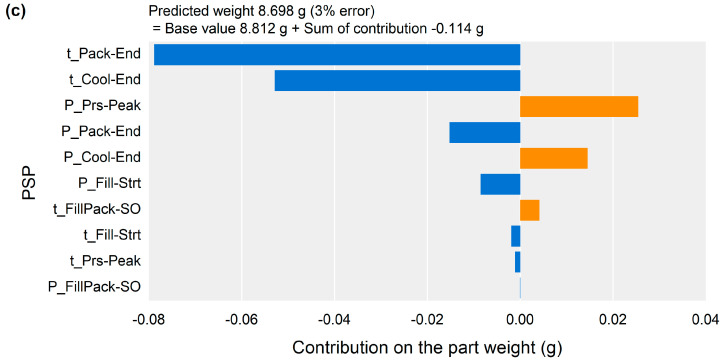
Contribution analysis results for the specific samples, (**a**) the highest-weight sample, (**b**) the middle-weight sample, and (**c**) the lowest-weight sample.

Figure 12 shows the contribution of each PSP to the part weight for the samples of the highest-, middle-, and lowest-weight samples. The highest weight could be attributed to the positive contribution of the time of the Pack-End and Cool-End PSPs, as shown in Figure 12a. The lowest weight could also be attributed to the contribution of the time of the Pack-End and Cool-End PSPs as shown in Figure 12c, and it showed a negative contribution instead. For the middle-weight sample shown in Figure 12b, the contribution of the pressure of the Pack-End PSP compensated for the effect of the time of the Pack-End and Cool-End PSPs. As a result of the contribution analysis for the specific sample, the time of the Pack-End and Cool-End PSPs, and the pressure of the Pack-End PSP were the most influential on the part weight. This result corresponding to the overall impact analysis results shown in Figure 9.

The contribution analysis results of the PSPs could be used to optimize the cavity pressure profile and the process parameters. The time and pressure of the PSPs to be adjusted could be determined by referring to these contributions. In the case of the lowest-weight sample shown in Figure 12c, the time of the Pack-End and Cool-End PSPs should be shifted to increase the part weight. The process parameters could be optimized to adjust the contributions of the time and pressure of the PSPs.

### 5.3. Validity Check

The interpretation of the cavity pressure profile using the proposed methodology is practically independent on prior knowledge and understanding. The simple flow pattern due to the simple cavity geometry results in the features of the cavity pressure profile having a simple relationship with the process parameters. The proposed methodology can be verified by comparing the process parameters with the PSPs from the cavity pressure profile determined by the process parameter settings. Consequently, the effect of the process parameters on the part weight is assumed to be transferred via the most influential PSPs.

**Figure 13:**
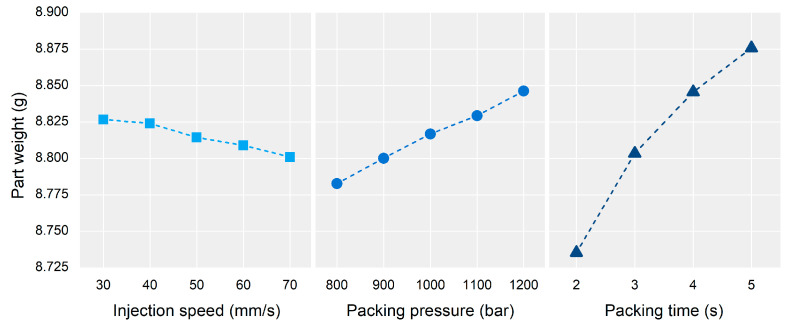
Effect of process parameters on the part weight.

Figure 13 shows the factorial plots representing the main effect of the process parameters on the part weight. Table 4 shows the ANOVA results. All the process parameters were significant factors affecting the part weight as the *p*-values of the process parameters were lower than 0.05. The most influential process parameter was the packing time, followed by the packing pressure. The effect of the injection speed was smaller than the process parameters related to the packing stage, and its effect on the part weight was negative.

**Figure 14:**
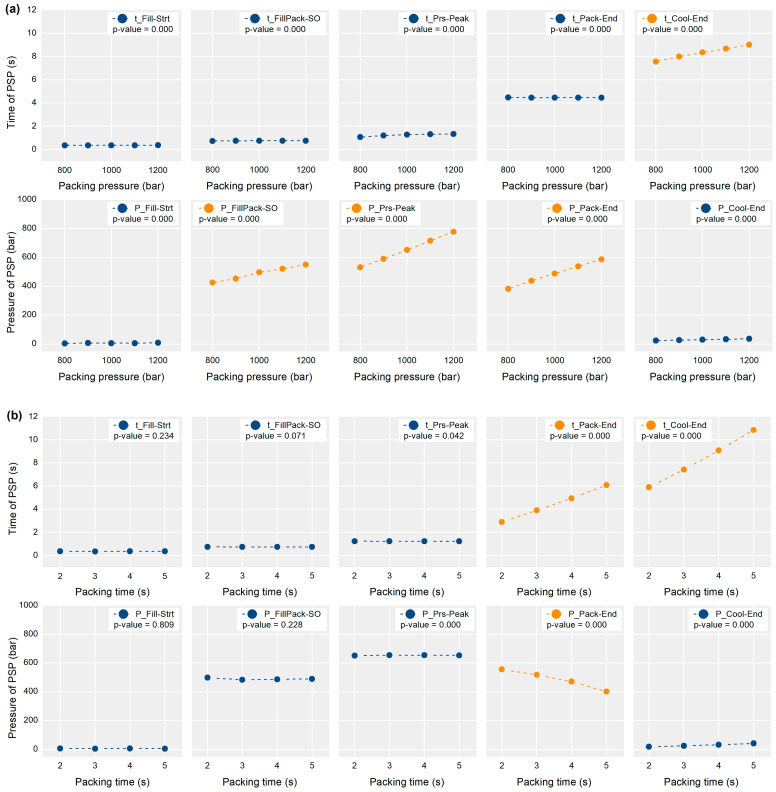
Effect of the influential process parameters on the time and pressure of the PSPs, (**a**) effect of packing pressure on the PSPs, and (**b**) effect of packing time on the PSPs.

Figure 14 shows the response of the time and pressure of the PSPs to the packing time and pressure. Each *p*-value from the ANOVA is shown in the legend. The packing pressure affected the pressure values of the FillPack-SO, Prs-Peak, and Pack-End PSPs, as shown in Figure 14a. The pressure values of the three PSPs are shown in the section after the filling. The pressure gradient from the nozzle of IMM to the cavity decreased because the melt flow stopped almost after the filling. Accordingly, the cavity pressure approached the packing pressure of IMM, the higher packing pressure directly increased the cavity pressure level after the filling. The pressure value of the Cool-End PSP was not affected by the packing pressure because the nozzle pressure could not be delivered due to the solidification of the melt delivery system. The time value of the Cool-End PSP was also affected by the packing pressure. The cavity pressure could be maintained for a longer time by higher packing pressure. The effect of the packing pressure on the part weight could be divided into the effect of the pressure value of the FillPack-SO, Prs-Peak, and Pack-End PSPs, and the time value of the Cool-End PSP. However, the pressure value of the FillPack-SO PSP had a small effect on the part weight because the entire cavity volume had not been filled at the moment of switchover.

The packing time affected the time values of the Pack-End and Cool-End PSPs, as shown in Figure 14b. A longer packing time resulted in a long-lasting packing stage in the cavity, shifting the time of the end of packing and cooling later. The pressure value of the Pack-End was negatively affected by the packing time. When the melt delivery system connecting the nozzle and the cavity was solidified during the packing stage, the cavity pressure slowly decreased even if the screw pushes the melt due to the packing pressure. This resulted in the cavity pressure value at the end of packing (Pack-End) having a negative response to the packing time. That is, the effect of packing time on the part weight could be divided into the effect of the time value of the Pack-End and Cool-End PSPs, and the pressure value of Pack-End PSP.

In summary, the effect of packing pressure and time was transferred by the four PSPs, namely the pressure of the Prs-Peak, Pack-End PSPs, and the time of the Pack-End and Cool-End PSPs. As Chen and Gao [48] and Xie et al. [49] pointed, the cavity pressure and the time duration during the packing and cooling stages were critical to the part weight as the shrinkage was compensated for by the cavity pressure during these stages. The impact analysis results (Figure 9) correspond to the ANOVA results and prior research results.

**Figure 15:**
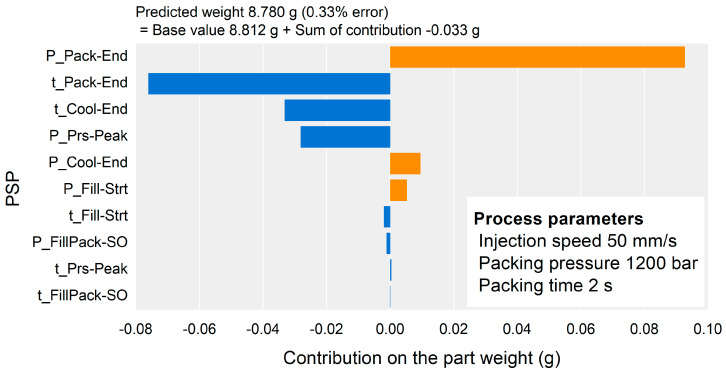
Contribution analysis result for the sample showing the contradictory effect of packing pressure and time.

The contribution analysis results for the specific sample corresponded to the relationships between the process parameters and the part weight. Figure 15 shows the contribution analysis result to the sample molded by the process parameters of the middle injection speed, highest packing pressure, and lowest packing time in Table 1. The pressure value of Pack-End related with the packing pressure shown in Figure 14a had the largest positive effect on the part weight. The time value of Pack-End related with the packing time shown in Figure 14b had the largest negative effect on the part weight. This result supported the validity of the proposed methodology.

## 6. Conclusions

A methodology to analyze the effect of the cavity pressure profile on part quality was proposed. The methodology analyzed the overall impact of the features in the cavity pressure profile on the part weight as part quality. The contribution of the features to a specific sample quality can be analyzed using this methodology. The methodology could be used to set the monitoring window for the cavity pressure profile and optimize the injection-molding process.

The methodology uses a machine learning model as a metamodel representing the relationship between the cavity pressure profile and part quality, and the interpretation method for the machine learning model. The PSPs as features of the cavity pressure profile were extracted from the time-series pressure data using the time-series segmentation algorithm. The time and pressure values of the PSPs were used to input data for the neural network, predicting the part weight as a quality index.

The simple MLP model represents the relationship between the PSPs and the part weight. The interpretation method of neural networks (SHAP) was used to analyze the trained neural network model. As a result of the model analysis, the cavity pressure profile could be analyzed using the overall impact of each PSP and the contribution of each to the specific sample. The impact analysis results represent the most influential feature of the part weight in the cavity pressure profile. The contribution analysis results propose the most influential feature of the cavity pressure profile to increase or decrease the specific part weight.

The analysis results of the effect of process parameters on both the part weight and the PSPs were compared to verify the proposed methodology. ANOVA showed that the packing time and pressure were the most influential process parameters. The effect of the packing time and pressure was transferred to the part weight through the four PSPs. This corresponds to the most influential features from the impact analysis results of the proposed methodology. The effects of packing pressure and time on the specific sample supported the contribution analysis results of the methodology.

The proposed methodology reduces the dependence on prior knowledge and understanding of the injection-molding process and polymer materials in order to analyze the cavity pressure profile. This methodology can be automated without human interference. The impact analysis results can be utilized to set an appropriate monitoring window for the cavity pressure profile. It is recommended that a monitoring window could be set to the most influential features, and its bounds should be adjusted based on the magnitude of the feature impact. The contribution analysis results can be used to optimize the cavity pressure profile. The feature points showing the largest contribution can be candidates for control points of optimization. Therefore, if the relationship between the process parameters and the response of the features is simple, the contribution analysis results can be used to optimize the process parameters. This approach can be used for important features such as cavity surface temperature and, melt temperature variation from the nozzle in injection molding as well as for other manufacturing processes.

The interpretation of the machine learning model used in this work contributes to a new framework for the monitoring and optimization of injection-molding processes. The methodology can be extended to the cavity surface temperature profile. The methodology can be used only for the cavity pressure profiles having similar shapes. To apply the methodology for the cavity pressure profiles showing various shapes such as the pressure in a sequential valve gated mold, the more flexible algorithm for extracting the PSPs should be applied. Future work will focus on the optimization of in-mold sensor profiles using the proposed methodology and a neural network as a metamodel for process parameters and PSPs.

## Figures and Tables

**Table 1:** Molding conditions.

Process Parameter	Levels	Values
Injection speed (mm/s)	5	30, 40, 50, 60, 70
Packing pressure (bar)	5	800, 900, 1000, 1100, 1200
Packing time (s)	4	2, 3, 4, 5

**Table 2:** Process state points.

Name	Meaning
Fill-Strt	Start point of filling stage
FillPack-SO	Switchover point from filling to packing stage
Prs-Peak	Maximum point of cavity pressure
Pack-End	Endpoint of packing stage
Cool-End	End of cooling stage

**Table 3:** MLP architecture.

Parameter	Value or Setting	Note
Number of layers	3	Single hidden layer
Input layer neurons	10	Time and pressure values of the extracted PSPs
Hidden layer neurons	8	Single digit number of neurons
Hidden layer activation	ReLU	[39]
Hidden layer initialization	He_Normal	[40]
Optimizer	Adamax	[41]
Loss for training	Mean square error	
Maximal training iteration	10,000 epochs	

**Table 4:** Analysis of variance (ANOVA) of process parameters for part weight.

Design Parameters	Degree of Freedom	Sum of Squares	Mean Square	F-Ratio	*p*-Value
Injection speed	4	0.027084	0.006771	133.20	0.000
Packing pressure	4	0.147199	0.036800	723.93	0.000
Packing time	3	0.834256	0.278085	5470.53	0.000
Error	200	0.010167	0.000051		
Total	299	1.044250			

## Data Availability

The data presented in this study are available on request from the corresponding author.
